# Tendon Transfer in Congenital Deficiency of Flexor Pollicis Longus

**Published:** 2017-01

**Authors:** Masoud Yavari, Ramin Farzam, Azade Riyahi

**Affiliations:** 1 Department of Hand Surgery, 15^th^ Khordad Hospital, Shahid Beheshti University of Medical Sciences, Tehran, Iran;; 2 Department of Occupational Therapy, Shahid Beheshti University of Medical Sciences, Tehran, Iran

**Keywords:** Flexor pollicis longus, Tendon transfer, Congenital aplasia

**DEAR EDITOR**

Congenital absence of flexor pollicis longus without associated anomalies is a rare cause of inability to flex the inter-phalangeal (IP) joint of the thumb. Other causes are: anomalous insertion of flexor pollicis longus (FPL), congenital tenovaginitis of the flexor tendon sheath, partial anterior interosseous nerve paralysis, traumatic rupture of FPL and anomalous bands connecting tendons.^1^

A three year old right handed girl was first observed by her parents to be unable to flex IP joint of her right thumb. The family history was negative for congenital anomalies. She had been operated for Hypertrophic pyloric stenosis when she was three weeks old. IP joint was hyper extended and active flexion was 0 degree and passive flexion was 30 degrees. No thenar muscle atrophy was noted. The patient was candidate for surgery with primary diagnosis of congenital trigger thumb.

The thumb was explored from base of the distal phalanx to distal of thenar muscles. The FPL was absent and a fibrous band was present instead. No pulley was present. Intra operative diagnosis was made and two stage re construction was planned. At first stage, a silicon rubber rod was inserted from zone 1 to zone 5. Then the fibrous band was cut proximally and formed as oblique pulley. The second stage of surgery was carried out by removing the flexor digitorum superficialis of ring finger. The IP joint was tensioned to 15 degree of flexion. Thumb spica cast was applied at the end of surgery. One month later, the cast was removed and rehabilitation by occupational therapist started. Fifty degree of flexion was gained after three months (Figure 1) and (Figure 2). 

**Fig. 1:**
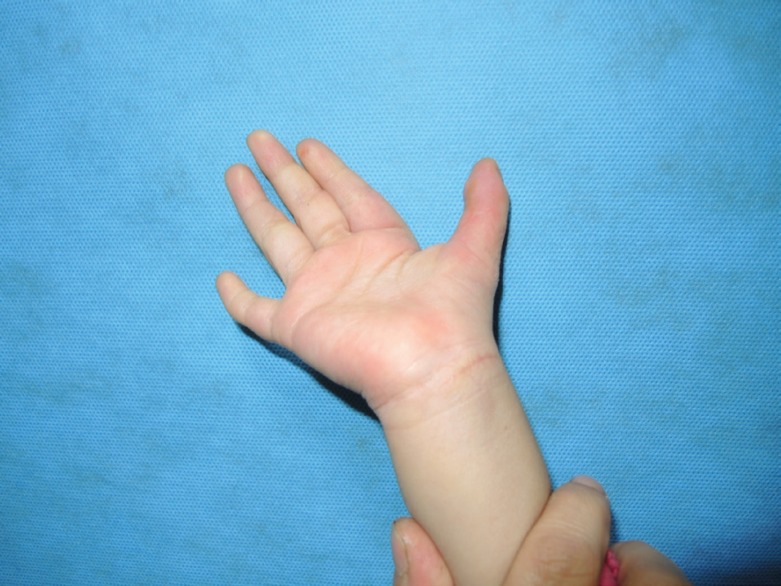
Thumb position at rest after surgery

**Fig. 2:**
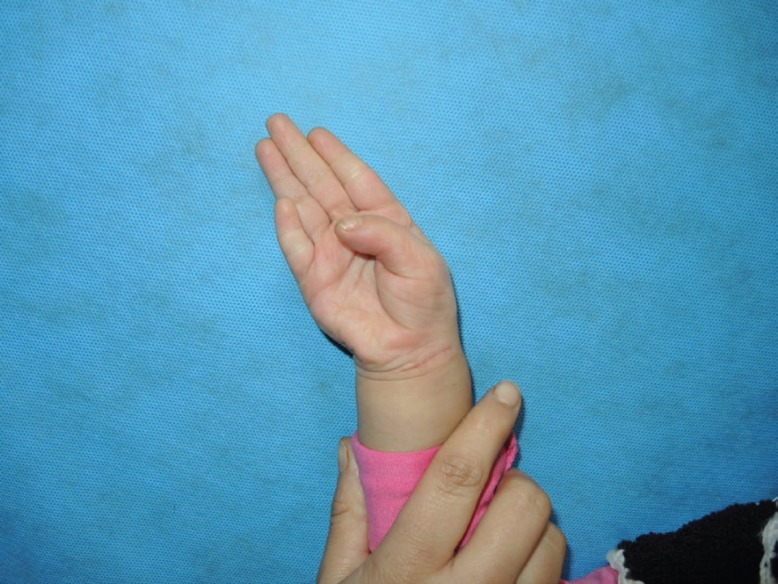
Thumb position actively flexed after surgery

The absence of FPL associated with aplasia of the thenar muscles was first described by Fromont in 1895.Uchida proposed a classification of absence of FPL with associated anomalies.^1^ They are divided into four groups: (i) Absence,^1^^-^^5^ (ii) Abnormal,^6^^,^^7^ (iii) Abnormal course,^1^ and (iv) Abnormal connection. Our case was type (i) which is a rare type.^1^ Clinically, the affected thumb is smaller than the other with a faint or absent flexion crease and restricted flexion at IP joint.

Ultrasound scans, routine X-rays and MRI nave been mentioned in various reports and investigations. Sonography is reported as an initial imaging modality and MRI to be considered as a complementary technique.^8^^-^^10^

Treatment options are one or two stage tendon transfer or fusion of IP joint, in functional position, in case of weakness or painful movements of the thumb. In our case, two stage tendon transfer was done and the result was good according to ´white´ criteria. So tendon transfer can be done at three years of age without fearing of adhesion or co-operation of the child. 

## CONFLICT OF INTEREST

The authors declare no conflict of interest.
